# Ro(a)d to New Functional Materials: Sustainable Isolation of High-Aspect-Ratio β-Chitin Microrods from Marine Algae

**DOI:** 10.3390/bioengineering12090969

**Published:** 2025-09-11

**Authors:** Jan Ludwig, Florian Kauffmann, Sabine Laschat, Ingrid M. Weiss

**Affiliations:** 1 Institut für Biomaterialien und Biomolekulare Systeme, Universität Stuttgart, Pfaffenwaldring 57, 70569 Stuttgart, Germany; 2 AMICA—Stuttgart Research Focus (SRF), Universität Stuttgart, Pfaffenwaldring 32, 70569 Stuttgart, Germany; florian.kauffmann@amica.uni-stuttgart.de; 3 Institut für Organische Chemie, Universität Stuttgart, Pfaffenwaldring 55, 70569 Stuttgart, Germany; sabine.laschat@oc.uni-stuttgart.de; 4 Stuttgart Research Center Systems Biology (SRCSB), Universität Stuttgart, 70569 Stuttgart, Germany

**Keywords:** β-chitin, chitin microrods, *Thalassiosira rotula*, sustainable biogenic nanomaterials, high aspect ratio, HAADF-STEM

## Abstract

High-aspect-ratio rod-shaped chitins such as chitin whiskers or chitin nano- and microfibers are particularly promising for a wide range of applications, including electrorheological suspensions, lightweight reinforcement material for biocomposites, biomedical scaffolds, and food packaging. Here, we report the first mild water-based mechanical extraction protocol to isolate β-chitin microrods from the marine algal species *Thalassiosira rotula* while preserving their structural integrity throughout the process. The resulting microrods could be distributed into two populations based on the fultoportulae from which they are extruded. The rods exhibit typical dimensions of 12.6 ± 4.0 µm in length and 75 ± 21 nm in diameter (outer fultoportulae) or 17.5 ± 4.7 µm in length and 170 ± 39 nm in diameter (central fultoportulae), yielding high aspect ratios of ~168 and ~103 on average, respectively. Due to this environmentally friendly extraction, the high purity of the synthesized chitin, and the renewable algal source, this work introduces a sustainable route to produce pure biogenic β-chitin microrods.

## 1. Introduction

Chitin, the homopolymer of β-(1⟶4)-linked *N*-acetyl-D-glucosamine (GlcNAc) units, is the most abundant polysaccharide in the marine environment, with annual productions estimated at ~2.8 × 10^7^ t–1.3 × 10^9^ t. It is therefore of prominent interest with regard to sustainable development and sea waste usage [1,2]. Yet despite this natural abundance and the rising global demand for sustainable high-performance materials, marine chitin remains still underexploited in technical applications compared to other polysaccharides [3].

In most biological systems, chitin occurs embedded within protein–mineral nanocomposites, rather than as a pure polymer [4,5,6,7]. These hierarchical chitin composites establish an important structure–function relationship, enabling diverse functions, for example, structural coloration in beetles, impact resistance of mantis shrimp dactyl clubs, and fracture toughness in mollusk shells [3]. However, this composite structure also complicates chitin isolation procedures [3]. Consequently, in recent years, there were significant research efforts to develop sustainable chitin extraction strategies from various sources [8,9,10,11]. Chitin nanomaterials already support a wide range of applications, including electrorheological fillers [12], adsorbents for toxic dyes [13], additives to reinforce foams and emulsions [14], and bioinks for 3D printing [9].

An exotic but promising source is algal chitin from species of the order *Thalassiosirales* (*Bacillariophyceae*). These organisms can pre-form microscaled β-chitin rods with high aspect ratios, typically exhibiting diameters of tens to hundreds of nm and lengths up to ~80 µm [15,16,17,18]. Because aspect ratios of chitin nanomaterials are a key parameter in material performance, this native rod geometry is especially interesting for downstream applications [19]. Traditionally, chitin is isolated from marine biomass that predominantly comprises crustacean shell waste, using top–down extraction strategies involving bulk demineralization (strong acids), deproteinization (strong bases), and decolorization (oxidants) steps [7,20]. While these pretreatments are effective in isolating pure chitin nanomaterials, they can alter key polymer parameters of the isolated chitin such as the degree of polymerization (DP), degree of acetylation (DA), and pattern of acetylation (PA) [21,22] and thereby interfere with native structure–function relationships [3]. Moreover, this generates substantial amounts of potentially hazardous chemical waste. After pretreatment, processing into nanochitin typically involves chemical or mechanical approaches [20]. Chemical acidic hydrolysis mostly relies on breaking down amorphous regions, producing lower-aspect-ratio chitin nanocrystals and nanowhiskers [20,23]. By contrast, mechanical processing (e.g., ultrasonication, high-pressure homogenization, or grinding) relies on the application of mechanical forces to disassemble the individual fibrils of chitin composites, yielding higher-aspect-ratio nanochitins, often termed nanofibers [11,19,20].

Since *Thalassiosira* spp. algae already possess the bio-machinery to synthesize chitin microrods in a pure form, no pretreatment is necessary. An interesting aspect of the *Thalassiosira rotula* system is that the chitin it produces can be modulated in vivo. With specifically tailored iminosugars made from inexpensive amino acid precursors, non-genetically modified *Thalassiosira rotula* algae were shown to produce chitin microrods with increased lengths compared to control conditions [15]. This makes them potentially valuable as chitin producers due to their ability to generate modified chitin without resorting to post-extraction chemical (dys-)functionalization. The ability to use these chitin-forming diatoms in photobioreactors is especially attractive regarding the biological upscaling of chitin rods [5].

By modifying the algal metabolism, a programmable route to sustainable production of tailored chitin nanomaterials is within reach [15,24,25,26]. A milder extraction procedure is, however, necessary to profit from these interesting nanochitins. Our aim, therefore, was to establish a purely water-based chitin microrod extraction method devoid of harsh chemical treatments, decreasing molecular and structural damage in the process. In the future, we hope that this extraction procedure will help to harvest in vivo modified algal chitin for use in downstream functional materials.

## 2. Materials and Methods

### 2.1. Thalassiosira Rotula Cell Culture

Cultures from the centric marine diatom *Thalassiosira rotula* were isolated from a marine sample gratefully obtained from the Alfred Wegener Institute (AWI) in Sylt, Germany. Generally, the cells were cultured with enriched artificial sea water medium (ESAW) [27,28], which was filtrated through a 0.2 µm filter under sterile conditions using a laminar flow hood before usage. The cells were incubated at 18 °C under a light intensity of 50 μmol photons per m^2^ per s following a 16 h:8 h light cycle (light from 6 am to 2 pm).

### 2.2. Chitin Microrod Isolation

For the microrod isolation, growth of a preculture of *Thalassiosira rotula* cells was initiated with at least 10 synchronized cells in 250 mL ESAW medium. The cells were grown until they reached a cell density of approximately 5000 cells/mL, which was achieved after ~5–7 days of culture. Afterwards, 50 mL of the pre-culture cell suspension was transferred to 950 mL fresh ESAW medium, which was then grown until a cell density of 10,000 cells/mL was reached (after ~7 days). The cell pellets were collected by centrifugation at 2000× *g*, for 5 min, at room temperature (RT). The pellets were washed twice with washing buffer (300 mM NaCl, 40 mM EDTA, pH 8.2) to remove any minerals and other ESAW residuals. Finally, the cell pellet was transferred into 2 mL microcentrifuge tubes and resuspended in fresh washing buffer. The cell suspension was vigorously shaken at 2000 rpm at 25 °C for 24 h (ThermoMixer^®^ C, Eppendorf, Hamburg, Germany) to dislodge the microrods from the algal cells. The next day, the suspension was filtered through a 6-well TC-insert (pore size 8 µm) (Sarstedt: 83.3930.800, Sarstedt AG & Co. KG, Nümbrecht, Germany) installed on top of a 50 mL Falcon tube via low-speed centrifugation (500× *g*, 3 min, RT) to isolate the free chitin microrods from the cells. The filtrate containing the fibers was transferred into another 2 mL microcentrifuge tube and washed five times with 2 mL double-distilled (dd) H_2_O, with centrifugation steps in between (15,000× *g*, 20 min, RT). Pellets with suspended rods were recovered, freeze-dried, and stored under vacuum at RT. The rod extraction was performed in at least three independent biological replicates to ensure reproducibility.

### 2.3. Light Microscopy

Light microscopy images were obtained in phase contrast mode using the Axiovert 200 M inverted light microscope (Carl Zeiss Microscopy GmbH, Jena, Germany). The microscope was equipped with a ×40 magnification objective lens (LD ACHROPLAN 40×/0.60, Carl Zeiss Microscopy GmbH, Jena, Germany) and a 98 CCD Camera (Zeiss AxioCam MRm, Carl Zeiss Microscopy GmbH, Jena, Germany) in combination with the Software Zen 2 blue edition (v2.0).

### 2.4. Electron Microscopy

Chitin microrods and *Thalassiosira rotula* cells were observed using a Zeiss EVO 15 scanning electron microscope equipped with Smart SEM software at 20 kV, 100 pA. The electrons were detected with a secondary electron detector.

For scanning electron microscopy (SEM) imaging, chitin microrods were resuspended in 100 µL ddH_2_O. Then, 10 µL of this suspension was carefully transferred onto a silicon wafer. For SEM imaging of *Thalassiosira rotula* cells, aliquots of 10 µL native living cells suspended in ESAW medium were transferred onto a 0.8 µm hydrophilic polycarbonate filter (Sigma Aldrich: ATTP02500, Isopore (Merck KGaA), Darmstadt, Germany). The formation of salt crystals during drying, which could rupture the cells, was prevented by gently removing the medium with vacuum-assisted filtration. This procedure did not require any fixation. The silicon wafers or polycarbonate filters were mounted onto aluminum SEM stubs topped with carbon Leit-tabs (12 mm diameter, Plano GmbH, Wetzlar, Germany). The rods and the cells were air-dried at RT and afterwards coated with Au/Pd in a sputter coater (Balzers MED 020, Leica, Wetzlar, Germany) for 60 s at 30 mA.

### 2.5. HAADF-STEM Microscopy

For high-angle annular dark-field scanning transmission electron microscopy (HAADF-STEM), 5 µL of a ddH_2_O suspension of isolated chitin rods was dropped on a 400-mesh copper grid coated with a carbon–formvar film (Plano GmbH, Wetzlar, Germany). Chitin rods of one *Thalassiosira rotula* chitin rod sample were negatively stained with 1% uranylacetate for 30 s for contrast enhancement. The TEM investigations were carried out in a ThermoFisher Spectra 300 at 300 kV. The TEM is equipped with a high-brightness Schottky Field Emission Gun (X-FEG). The STEM images were recorded with an HAADF detector using a dwell time of 10 µs. The camera length was set to 115 mm and the convergence angle to 22.5 mrad.

### 2.6. Imaging and Statistical Analysis of the Chitin Microrods

Image analysis was performed using ImageJ v1.54p [29]. For the generation of histograms of width and length distributions, randomly, 100 chitin rods were selected from SEM images and were measured manually using the line tracing feature of ImageJ. Histograms and boxplots were generated using R statistical software (v 4.4.2) [30] in combination with ggplot2 (v 3.5.2) [31] and the mclust (v 6.1.1) [32] package for the Gaussian mixture modeling approach to analyze bimodal distributions.

## 3. Results and Discussion

### 3.1. Native Chitin Rod Formation in Thalassiosira rotula

It has been shown that iminosugars are able to modulate chitin synthesis in vivo, increasing the lengths of chitin rods compared to control conditions [15]. To investigate these chitin rods for developing applications, we wanted to establish a procedure that allowed mild extraction for a rigorous structural and chemical analysis aiming at preserving the native structure–function relationship of these chitins. We first investigated intact *Thalassiosira rotula* cells for their native rod geometries using light and electron microscopy to provide a baseline for subsequent chitin rod extraction. This allows us to define the target rod morphology that we want to preserve during the extraction procedure.

**Figure 1:**
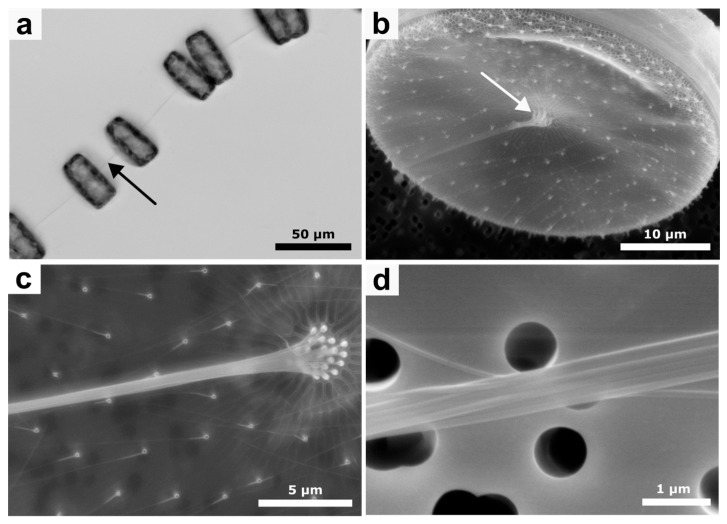
*Thalassiosira rotula* cells and chitin microrods. (**a**) Light microscopy images of *Thalassiosira rotula* cells connected by extracellular chitin rod bundles (black arrow). (**b**) SEM image of a *Thalassiosira rotula* cell showing the central chitin rod formation apparatus (white arrow). (**c**) Magnification of the area around the central chitin rod formation apparatus consisting of multiple central fultoportulae, as well as the outer fultoportulae distributed on the biosilica valve surface. (**d**) Higher magnification of one of the *Thalassiosira rotula* composite chitin fibers consisting of bundles of individual rods originating from central fultoportulae, highlighting the hierarchical properties of diatom chitin fibers.

Live cell imaging using light microscopy showed that *Thalassiosira rotula* cells are connected together by single extracellularly formed chitin rods (Figure 1a). Further inspection via electron microscopy (Figure 1b–d) demonstrated that each observed chitin rod consisted of a bundle of parallel micro- or nanorods. Figure 1b,c show the biosilica valve of *Thalassiosira rotula*. Distributed on its surface, some protruding specialized biosilica pores (fultoportulae) are visible. Chitin synthases located in the membrane underlying the fultoportulae are responsible for forming the individual chitin rods. Notably, the rods synthesized by the central fultoportulae are connected together, forming a microrod bundle. At higher magnifications (Figure 1d), individual rod bundles appear smooth, straight, and uniform in thickness. The number of fultoportulae in the center, as well as the smaller ones distributed on the valve, are not consistent among the cells but tend to comprise around 15 to 17 central fultoportulae and 101–117 outer fultoportulae (Table 1). Measurement of the chitin rod diameters showed that they are different based on the position of the fultoportulae from which they originated. Chitins from central fultoportulae formed thicker chitin rods with 111 ± 33 nm on average, while outer fultoportulae extrude thinner rods with an average of 69 ± 18 nm. The rod diameter is probably limited by the diameter of the silica pore itself (216 ± 44 nm for the central fultoportulae vs. 165 ± 33 nm for the outer fultoportulae). Collected morphological measurements of the native synthesized rods and of *Thalassiosira rotula* fultoportulae are provided in Table 1.

After having defined the native *Thalassiosira rotula* rod geometries, a water-based rod extraction workflow was designed for mechanically removing the microrods from fultoportulae while minimizing rod damage.

### 3.2. Thalassiosira rotula Chitin Rod Isolation

Isolation of structurally intact β-chitin microrods required cultivation of *Thalassiosira rotula* diatoms under controlled conditions to maximize rod production while minimizing cellular aggregation. We started cultivation with a preculture of a synchronous population of at least 10 cells to reduce the variability of the timing of chitin rod extrusion, which happens once per day on average [15]. Cultures were then grown at a 1 L scale in ESAW medium until they reached a density of around 10,000 cells/mL but were not cultivated for more than 7 consecutive days in the same medium. From experience, prolonging the culture beyond this point led to nutrient depletion, cell clumping, and stagnated growth, coinciding with increased biofilm formation [33]. Harvesting the chitin rods in this state complicated the purification procedure at the end.

Cells were harvested by low-speed centrifugation to minimize shear and to prevent cell lysis, limiting the unwanted spill of cellular contents into the suspension. Afterwards, the pellet was washed twice with 300 mM NaCl and 40 mM EDTA (pH 8.2). The washing step served two purposes: (1) removal of residual medium components; (2) maintenance of osmotic balance to minimize lysis. The pH was selected to resemble natural seawater at pH 8.2 [34] and to exploit potential self-assembly of β-chitin, as previously reported for squid pen chitin at pHs between 7.0 and 8.5 [35]. Experimental variations in NaCl and EDTA concentrations at different pH values demonstrated that the rods and the cells remained structurally intact as determined by light microscopy until a pH of 10, whereas extreme alkaline conditions at pH 13 caused dissolution of the silica frustule, interfering with the extruded chitin rod isolation.

After washing, the cell pellet (~10 mg/mL on average per batch) was subjected to overnight mechanical shaking treatment at 2000 rpm. This treatment proved to be effective in dislodging the microrods from fultoportulae requiring no further treatment. Previous reports on top–down mechanical approaches showed that high aspect ratios of chitin nanomaterials can be preserved [19,23]. Subsequently, we separated the rods from the cells by filtration through 8 µm filters. Given that typical *Thalassiosira rotula* valve diameters are around 25 µm (Figure 1b), this cutoff retained cells on the membrane while allowing the microrods to pass through. The chitin rod-containing filtrate was then subjected to multiple washing steps using ddH_2_O to remove remaining salts from the washing buffer, yielding a mostly pure pellet (5 mg/L ESAW medium) of chitin rods suitable for imaging and analysis. We compiled state-of-the-art methodologies to extract chitin from diatoms over the years in Table 2 to contextualize our approach.

Extraction of chitin from algae requires the distinction of two separate chitin fractions. For both of them, various extraction procedures are described in the literature (Table 2): (1) bulk chitin, which is associated with the silica cell wall, and (2) chitin microrods that are extruded from fultoportulae into the extracellular environment. Harvesting chitin embedded into the cell wall requires the dissolution of the biosilica frustule. This is typically achieved by treatment of cells with harsh bases such as KOH, exploiting the high pH environment [37], or using reagents such as hydrofluoric acid or ammonium fluoride, as performed in [37,38,40], followed by repeated washing and drying cycles.

Isolation of extruded rods, however, typically employs mechanical approaches including the use of blending [16,36,39] or centrifugation [17,37,40]. Since we were interested exclusively in the chitin rods extruded from the cells and not present in cell walls, our initial starting point was based on a mechanical procedure. However, while approaches such as short bursts of blending or centrifugation work well for algal species that form single chitin rods (*Thalassiosira fluviatilis Hustedt* [36], *Thalassiosira weissflogii* [37,39,40], or *Cyclotella cryptica* [16,17]), *Thalassiosira rotula* also produces bundled chitin microrods (Figure 1b–d) that proved to be resistant using these approaches. Longer blending times led to cell rupture, which made it more difficult to isolate chitin in the further steps, and centrifugation in general did not result in effective chitin rod extraction at all in *Thalassiosira rotula*. Thus, we decided to use controlled shaking overnight to efficiently dislodge the chitin rods without rupturing the cells in the process.

In the development of the chitin pellet purification steps, we wanted to preserve the structure–function relationship of the native chitin. Therefore, our processes were all carried out at room temperature, and we abstained from harsh chemicals such as strong acids, bases, or bleaches. For example, in [17], the extruded cell pellet was dried at 200 °C. In contrast to our method, this drying process might damage the native structure. Treatments of chitin pellets with KOH or NaOH as in [37,39,40], with HCl as in [16,37,40], and with oxidants for decolorization as in [37,39,40] could be avoided. In summary, it turned out that the mild extraction method presented here is inspired by a methodology dating back, to the best of our knowledge, to 1977, which was developed with a clear focus on structural preservation.

### 3.3. Electron Microscopical Analysis of the Isolated Chitin Rods

**Figure 2:**
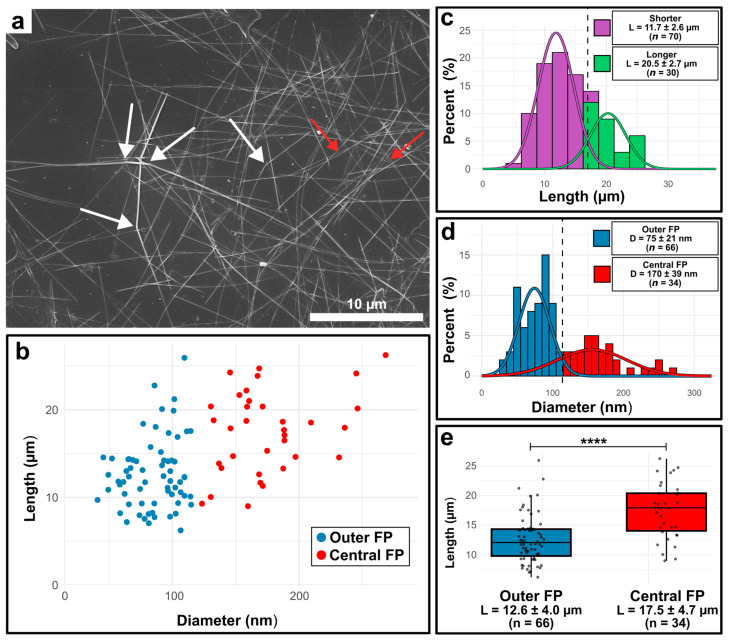
Electron micrograph of chitin rods from *Thalassiosira rotula* isolated with the water-based mechanical extraction method and statistical evaluation of extracted chitin rods. (**a**) Exemplary SEM image of a rod sample. White arrows point to instances where rods bend, and the red arrows point to locations where the rods unwind, showing that they constitute multiple fibrils. (**b**) Scatterplot of length and diameter of *n* = 100 individual rods measured from multiple SEM images. Gaussian mixture modeling (mclust) was used to classify rods into two populations corresponding to synthesis from outer (blue) and central (red) fultoportulae (FP). (**c**) Bimodal distribution of chitin rod lengths. Classification by Gaussian mixture modeling (mclust) resulting in two populations corresponding to shorter (purple) and longer (green) chitin rods. Mean values and standard deviation are provided in the legend. The dotted line represents the cutoff of 16.95 µm. (**d**) Bimodal distribution of chitin rod diameters separated into outer (blue) and central (red) fultoportula populations. Solid curves represent Gaussian fits calculated with the mclust package in R. The dotted line represents the cutoff of 107 nm between the two populations determined by Gaussian mixture modeling. Mean values and standard deviation are provided in the legend. (**e**) Rod length distributions divided into FP populations. Mean lengths of the two populations are provided beneath the boxplots. *p*-values were calculated using a two-sample *t*-test in R (**** = 3.1 × 10^−6^).

We quantified rod geometries after the water-based extraction procedures to assess whether they retained the native architecture observed in live cells (Table 1). Rod lengths and diameters were measured from scanning electron microscopy images. Figure 2a shows an exemplary SEM image of air-dried chitin microrods deposited on a silicon wafer. Longer rods displayed a degree of bending (Figure 2a, white arrows) and an occasional partial unwinding into smaller rods was observed, hinting at an underlying fibrillar structure, as is common among chitin materials (Figure 2a,b, red arrows) [3]. We measured the diameters and lengths of *n* = 100 randomly selected microrods across multiple SEM images. A scatterplot of the measurements is provided in Figure 2b, while the corresponding distributions of length and diameter are shown as histograms in Figure 2c and 2d, respectively. Because we observed bimodal distribution patterns of length and diameter, we performed Gaussian mixture modeling using the mclust package in the R environment to separate the two populations of rod diameters and rod lengths to evaluate them. Regarding rod lengths, we could separate the rods into longer and shorter populations (Figure 2c). Longer chitin rods possessed a mean length of 20.5 ± 2.7 µm (*n* = 30) on average, whereas the shorter rods reached a mean length of 11.7 ± 2.6 µm (*n* = 70) on average. We also determined mean diameters of 75 ± 21 nm (*n* = 66) for population 1 and 170 ± 39 nm (*n* = 34) for population 2, with a cutoff determined to be 107 nm (Figure 2d). The two diameter populations are indicated accordingly in the scatterplot as well (Figure 2b). When compared with native microrod measurements from Table 1, the two distinct diameter populations map onto the rods extruded from the different types of fultoportulae. From outer fultoportulae, generally thinner rods are extruded (69 ± 18 nm), whereas central fultoportulae were measured to extrude rods with average diameters of 111 ± 33 nm. The native rods being thinner compared to the extracted rods was probably due to biological variations over the time of the experiments. However, a succinct trend is observable between thinner and thicker rod geometries, and we therefore determined these two different populations to be the rods extracted from outer and central fultoportulae. A predominant extraction of rods with lower diameters is expected, since the outer fultoportulae outnumber the central ones by a factor of 6.8. Motivated by the bimodal distribution of the rod lengths, we also compared the lengths between rods classified as from “outer” and “central” fultoportulae (Figure 2e). Comparisons revealed clear differences between the groups, with rods from outer fultoportulae being primarily shorter (12.6 ± 4.0 µm, *n* = 66) compared to the rods in the central fultoportula population (17.5 ± 4.7 µm, *n* = 34). Statistical evaluation using a two-sample t-test confirmed that these differences were significant (*p* = 3.1 × 10^−6^). When considering the two fultoportula groups, the rod geometries yield high aspect ratios (*L*/*d*) of ~168 (outer fultoportulae) and ~103 (central fultoportulae). Furthermore, the broad length distribution (5–20 µm for rods originating in outer fultoportulae and 15–30 µm for central fultoportulae) is not surprising, since at the time of harvesting, rod synthesis is not synchronized anymore. We tried to combat this effect by initiating the culture with synchronous cells. However, maintaining perfect synchrony remained difficult to achieve after several days of culture.

**Figure 3:**
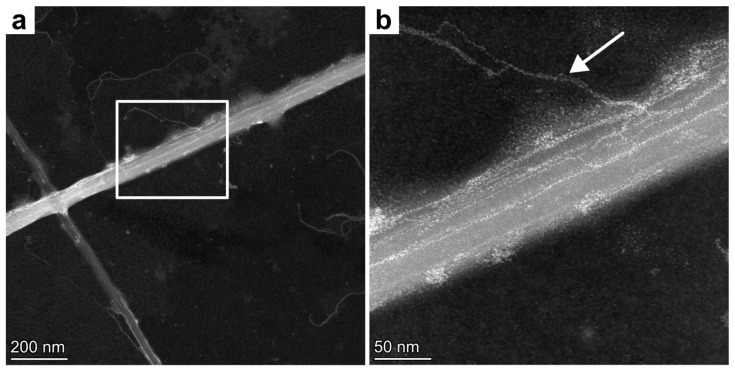
HAADF-STEM images of the chitin rods isolated from *Thalassiosira rotula* using the water-based extraction procedure, showing a nanofibrillar structure in the chitin rods. (**a**) Two chitin rods crossing. (**b**) Magnification of (**a**). The white arrow points to a secondary nanofibril protruding from the main rod.

We performed high-angle annular dark-field scanning transmission electron microscopy (HAADF-STEM) on uranylacetate-stained chitin rods to examine the ultrastructure of the microrods in more detail. This technique allows the observation and confirmation of the ultrastructural integrity of the chitin rods isolated with the water-based extraction method. In Figure 3a, two chitin microrods are shown, ~75 nm and ~50 nm in diameter. This corresponds to chitin rods extruded from outer fultoportulae (Figure 2). In contrast to SEM (Figure 2), an internal texture is visible in the HAADF-STEM image (Figure 3). We interpret this as an additional hierarchical level. It is interesting to note that even chitin rods originating from smaller fultoportula populations consist of several fibrils.

The rods seem to consist of several nanorods, which measure between 16 and 20 nm on average. Higher-magnification imaging (Figure 3b) reveals some secondary nanofibrillar structures protruding from one of the main rods (white arrow). These nanofibrils are thin, approximately 4.2–4.5 nm in diameter, and seem to be more flexible than the main rod and possibly wind around the main fibril.

### 3.4. Comparison with Nanochitins from Other Sources

To our knowledge, this is the first report of the extrusion of chitin microrods from *Thalassiosira rotula* under water-based extraction conditions. Most nanochitin studies focus on biomass typically consisting of crustacean shell waste [4,7] or squid pen β-chitin sources, where substantial pretreatment is necessary to remove minerals, proteins, and pigments [7]. Such pretreatments may alter the DP, DA, and PA and therefore disrupt the native structure of chitin [3,22]. In contrast, our mechanical water-based workflow retains the native high-aspect-ratio rod geometry and molecular composition by the elimination of harsh chemical treatments.

In Table 3, we summarize representative mechanical nanochitin preparations across α- and β-chitin sources, together with the dimensions of the resulting nanochitins. Notably, due to an overwhelming amount of extraction procedures and possible chitin sources, the research on nanochitins is relatively unfocused and thus difficult to compare, especially considering that we use a rather exotic source for β-chitin nanomaterials. However, as a general trend, it can be stated that α-chitin sources lead to lower-aspect-ratio (*L*/*d*) nanochitins (mostly <100) compared to β-chitin sources, which have higher aspect ratios. The chitin microrods we isolated from *Thalassiosira rotula* exhibited aspect ratios of ~168 for rods extruded from outer fultoportulae and ~103 for rods extruded from central fultoportulae. Both populations of rods show high aspect ratios comparable with other sources of chitin nanomaterials. Rods from outer fultoportulae show similar if not higher aspect ratios compared to chitin rods isolated from squid pen, while differences in rod geometries are expected to translate into variations in bending stiffness and persistence length [19,41,42,43,44]. However, considering only diameters, squid pen nanochitins are typically thinner. This is compensated for by the length of *Thalassiosira rotula* rods with 12.6 and 17.5 µm on average (this study) dominating the length scale among all the materials considered in Table 3.

Interestingly, though, chitin rods from *Thalassiosira rotula* generally are quite short when compared with microrods isolated from other species of *Thalassiosirales* (Table 2), which often lie in the range of 50–80 µm. In a previous study, we observed chitin rods synthesized in vivo between 19 and 23 µm for *Thalassiosira rotula* [15]. These deviations probably reflect the biological variations among different cell populations over time.

Our ultrastructure investigations using HAADF-STEM (Figure 3) show for the first time the internal fibrillar structure of *Thalassiosira rotula* chitin nanorods. The occasionally protruding chitin nanofibrils, which were measured as 4.2–4.5 nm in diameter, are consistent with diameters of β-chitin microrods from squid pen in Table 3, supporting the view that *Thalassiosira rotula* microrods are composed of hierarchically ordered β-chitin fibrils.

These comparisons especially underline two advantages in the use of *Thalassiosira rotula* as a source of chitin rods for high-aspect-ratio materials. First, pre-formed chitin microrods simplify the isolation procedure, since pretreatment steps can be avoided. Second, in other studies, it was shown that *Thalassiosira rotula* chitin rods can be modulated in vivo [15,25,26]. Given that rod geometry is important for material performance in applications such as mechanical reinforcements, foam stabilization, and electrorheology, the coupling of biological tuning with a mild extraction procedure that preserves the structure–function relationship provides a promising route to programmable sustainable chitin building blocks. We note that upscaling of species of other *Thalassiosirales* microalgae was shown to be possible in photobioreactors [5], suggesting that a scale-up of *Thalassiosira rotula* cultures is feasible after some optimization. Based on a cost of 55 USD/L of enriched artificial sea water medium (UTEX, 2025 [49]) and assuming suitable infrastructure, our extraction procedure corresponds to a production cost of approximately USD 11 per mg of high-aspect-ratio chitin rods (excluding electricity costs). Chitin yields, however, vary greatly between species. For example, *Cyclotella cryptica* has been reported to produce up to 316 mg chitin rods per liter [5], corresponding to 0.17 USD/mg chitin rods. Nevertheless, such species have not been evaluated for their capacity to undergo chitin modulation or for their potential to produce different fractions of chitin rods.

## 4. Conclusions

In this study, we successfully developed a water-based extraction procedure for β-chitin microrods from *Thalassiosira rotula* cells. The method avoids harsh chemical treatments, preventing the release of potential hazardous chemicals into the environment, while minimizing damage, to preserve the native hierarchical chitin structure of the microrods. SEM imaging confirmed the structural integrity and the high aspect ratios of the microrods (~168 and ~103 for rods synthesized by outer and central fultoportulae, respectively).

Our HAADF-STEM analysis revealed for the first time the underlying hierarchical fibrillar composite structure of algal β-chitin rods and showed occasionally protruding nanofibrils not visible using SEM investigations. This sustainable procedure provides the foundation for analyzing and understanding rod morphologies from microrods synthesized under chitin-modulating conditions. We acknowledge that a yield of 5 mg chitin rods per liter of medium is insufficient for technologies that require bulk chitin. However, we hope that in the future, chitin nano- and microrods extracted using this procedure will be applied for a wide range of specialized applications, e.g., as potential fillers for electrorheological suspensions, and lightweight reinforcement material for biocomposites, biomedical scaffolds, or food packaging.

## Figures and Tables

**Table 1:** Morphological data collected on *n* (number of) *Thalassiosira rotula* cells. The averages were calculated based on measurements of multiple SEM images. The error is the standard deviation calculated from the measured values.

	Central Position	Outer Position
Number of fultoportulae per valve (#)	16.1 ± 1.2 (*n* = 12)	109.0 ± 8.5 (*n* = 4)
Fultoportula diameter (nm)	216 ± 44 (*n* = 35)	165 ± 33 (*n* = 68)
Chitin rod diameter (nm)	111 ± 33 (*n* = 34)	69 ± 18 (*n* = 50)

# signifies the fultoportulae numbers.

**Table 2:** State-of-the-art-methods of chitin extraction from diatoms used over the years in publications. For details, see the references listed herein.

Algal Chitin Source	Method Used to Extract Chitin Rods	Year of Publication	Average Rod Measurements		Reference
			Length (µm)	Diameter (nm)	
*Thalassiosira fluviatilis Hustedt* (extruded chitin)	Dislodging chitin with Waring blender for 1–2 s, removing the cells with Sharples continuous flow centrifugation of supernatant at 2/3 maximum speed, filtration of chitin-rich supernatant through 1.2 µm membrane filter; chitin formed a “mesh” on the filter, which was water-washed, air- or oven-dried at 40 °C, separated from the filter by scraping off the mesh, water-washed, treated with MeOH or ether, and dried under vacuum over P_2_O_5_	1965	60–80	100–200	[36]
*Cyclotella cryptica* (extruded chitin)	Collection of algal cell pellet by centrifugation, vacuum-assisted filtration of the supernatant to collect chitin “mesh” on membrane, 5×water wash on the filter, scraping off chitin “mesh” and wash 2× with ethanol, collection by centrifugation and drying (15 min at 100 °C or 200 °C)	1977	50	5–30	[17]
*Thalassiosira weissflogii* (cell wall and extruded chitin)	Cell collection via centrifugation, treatment of the pellet with 5% KOH (overnight, RT), methanol (80 °C, 2 h), 0.34% NaClO_2_ (pH 4, 70 °C, 6 h), 0.1 N HCl (boiling, 1 h), 1% HF (RT, overnight) rinsing with water after each step, lyophilization, storage under vacuum	2003	Not provided	Not provided	[37]
*Thalassiosira pseudonana* (cell wall chitin)	Cell walls harvested by high-speed centrifugation in a Westfalia separator or filtration on nylon filters, twice boiling of the pellet in 0.1 M EDTA and 2% SDS, centrifugation and water wash until supernatant was colorless, lyophilization overnight. Dissolution of silica frustules using 8M NH_4_F/2M HF (RT, pH 4–5, 20 min), centrifugation, 4× water wash, lyophilization overnight. Treatment with 2.5 M NaOH (37 °C, 2 h), centrifugation, 4× water wash, lyophilization overnight	2009	Not provided	25	[38]
*Thalassiosira weissflogii* (extruded chitin)	Dislodging fibers from algal cells by blending in a kitchen mixer for several seconds, low-speed centrifugation and collection of chitin-rich supernatant, high-speed centrifugation to obtain chitin-rich pellet, treatment with 1 N KOH overnight at RT, 0.3% NaClO_2_ (pH 4.8, 80 °C, 3 h) repeated three times with water washing between each step	2011	Not provided	29.8	[39]
*Cyclotella* sp. (extruded chitin)	Dislodging of chitin using a Waring blender, low-speed centrifugation and collection of supernatant, high-speed centrifugation to obtain chitin-rich pellet, HPLC-grade water wash, treatment with 1M HCl at 70 °C (30 min), 0.5% (m/m) SDS, 95% EtOH (RT), air-drying at 45 °C for 4 h	2019	60	56	[16]
*Thalassiosira weissflogii* (cell wall and extruded chitin)	Cell collection via centrifugation, treatment of cell pellets and supernatant with methanol (65 °C, 2 h), 5% KOH (RT, overnight), 0.34% NaClO_2_ (70 °C, 6h), 0.1 N HCl (boiling, 1 h), 1% HF (RT, overnight) with high-speed centrifugation steps and removal of supernatant in between. Sample was dried at 80 °C, stored at −80 °C	2023	Not provided	Not provided	[40]

**Table 3:** Preparation of nanochitin from different sources via mechanical extraction procedures.

Chitin Source	Chitin Polymorph	Length [µm]	Diameter [nm]	Aspect Ratio (*L*/*d*)	Reference
Squid pen (*Illex argentinus*)	β	1–3	14 ± 7	~143 (up to 750)	[41]
Squid pen (*Todarodes pacificus*)	β	>1	3–4	>250	[42]
Squid pen (*Loligo bleekeri*)	β	0.48	4.1	~117	[43]
Squid pen (*Illex argentinus*)	β	1.73 ± 0.59	17.24 ± 2.02	~100	[44]
Algae (*Phaeocystis globosa*)	α	3	37 ± 8	~81	[43]
Crab	α	0.25 ± 0.14	6.2 ± 1.1	~40	[45]
Lobster (*Homarus americanus*)	α	0.697–1.167	3.1–3.5	199–376	[46]
Lobster (*Cervimunida johni*)	α	5	80–100	>50	[47]
Fresh speckled swimming crabs (*Arenaeus cribrarius*)	α	5–10	80–100	>50	[48]

## Data Availability

The raw measurements (length, diameter, and aspect ratio) and the raw scanning electron microscopy images for each individual rod measurement in the current study are available in the data repository of the University of Stuttgart (DaRUS) under https://doi.org/10.18419/DARUS-5312.

## Abbreviations

The following abbreviations are used in this manuscript:

AWI	Alfred Wegener Institut
DP	Degree of polymerization
DA	Degree of acetylation
ddH_2_O	Double distilled water
ESAW	Enriched artificial sea water
FP	Fultoportulae
GlcNAc	*N*-acetyl glucosamine
HAADF-STEM	High-angle annular dark-field scanning transmission electron microscopy
PA	Patterns of acetylation
RT	Room Temperature
SEM	Scanning electron microscopy
